# Cytokine-induced killer (CIK) cells, successes and challenges: report on the first international conference dedicated to the clinical translation of this unique adoptive cell immunotherapy

**DOI:** 10.1007/s00262-023-03605-1

**Published:** 2024-01-27

**Authors:** Amit Sharma, Xiubao Ren, Antonio Rosato, Dario Sangiolo, Zibing Wang, Sarah Tettamanti, Yi Zhang, Eva Rettinger, Kevin Aaron Fenix, Roberta Sommaggio, Elisa Cappuzzello, Ingo G. H. Schmidt-Wolf

**Affiliations:** 1 Department of Integrated Oncology, Center for Integrated Oncology (CIO) Bonn, University Hospital Bonn, Bonn, Germany; 2 Tianjin Medical University Cancer Institute and Hospital, National Clinical Research Center for Cancer, Tianjin, China; 3 Department of Surgery, Oncology and Gastroenterology, University of Padova, Padua, Italy; 4 Department of Oncology, University of Turin, Turin, Italy; 5 Immunotherapy Department, Affiliated Cancer Hospital of Zhengzhou University and Henan Cancer Hospital, Zhengzhou, China; 6 Tettamanti Center, Fondazione IRCCS San Gerardo dei Tintori, Monza, Italy; 7 Biotherapy Center, The First Affiliated Hospital of Zhengzhou University, Zhengzhou, China; 8 Frankfurt Cancer Institute (FCI), Goethe University, Frankfurt am Main, Germany; 9 Discipline of Surgery, Adelaide Medical School, The University of Adelaide, Adelaide, SA Australia; 10 Immunology and Molecular Oncology Unit, Veneto Institute of Oncology IOV - IRCCS, Padua, Italy

**Keywords:** Cytokine-induced killer (CIK) cell therapy, Cancer, Immunotherapies

## Abstract

On August 30, 2023, experts from Germany and abroad met to discuss the successes and challenges of cytokine-induced killer cell (CIK) therapy, that recently celebrated its 30th anniversary providing treatment for cancer. This first virtual conference was hosted by CIO Bonn, a certified Comprehensive Cancer Center (CCC) funded by German Cancer Aid (DKH). In addition to keynote speakers involved in CIK cell clinical trials or optimized preclinical models to improve this adoptive cell immunotherapy, more than 100 attendees from around the world also participated in this event. Initiatives to establish the International Society of CIK Cells (ISCC) and a stronger CIK cell network guiding preclinical research and future clinical trials were also announced.

## Introduction

The concept of isolating immune cells, expanding their numbers, modifying them when necessary, and overall enhancing their anticancer capabilities is the mainstay of cancer immunotherapy. Cancer immunotherapies have become mainstream in current cancer treatment and are fully integrated into standard therapies. Of these approaches, the successful implementation of cytokine-induced killer (CIK) cell therapy, an adoptive cell therapy, is clearly evident in the clinical settings of cancer [1]. A pioneering work of CIK cell generation was conducted by Schmidt-Wolf and colleagues in 1991 [2], and soon the clinical applicability was confirmed (in 1999) for the treatment of cancer patients [3]. Over the past 3 decades, numerous clinical trials and preclinical evaluations have been conducted, and thousands of patients have benefited from this approach. Since CIK cells can be easily expanded from peripheral blood lymphocytes with a cocktail of stimuli (IFN-γ, anti-CD3 antibodies, IL-2, IL-1β), they remain one of the most popular and reliable options for cancer immunotherapy. Furthermore, CIK cells are heterogeneous in nature and can be easily characterized phenotypically, as they are mainly composed of CD3+CD56− (T cells), CD3+CD56+(NKT cells) and CD3−CD56+ (NK cells) subpopulations, all of which have a strong impact on target cells. Meanwhile, CIK cell treatment has been approved in many countries, including Germany. To celebrate this and to discuss future possibilities, this first virtual conference was hosted by Prof. Ingo Schmidt-Wolf (currently serving as Director, Department of Integrated Oncology, Center for Integrated Oncology Bonn, University Hospital Bonn, Bonn, Germany) and organized by CIO Bonn, a certified Comprehensive Cancer Center (CCC) funded by German Cancer Aid (DKH).

After welcoming remarks, *Prof. Schmidt-Wolf (Germany)* shared the initial challenges his team faced in culturing and optimizing the in-vitro killing ability of CIK cells. Also, how they succeeded in confirming the tumor-killing ability of CIK cells first in a SCID mouse lymphoma model and later in the clinical treatment of cancer patients. He acknowledged the efforts made by the research community to identify the mechanisms underlying the antitumor activity of CIK cells, noting that all involved agreed with the conclusion that CIK cells exert their cytotoxicity primarily through the interaction between NKG2D, leading to the secretion of cytokines such as IFN-gamma. Nevertheless, he believes that there are other, as yet unknown, ways that CIK cells can be used to fight cancer cells, which the research community will surely witness in next years. He also emphasizes that the success of CIK cells is partly due to the enrichment of immune cells (T cells, NK cells, NK T cells), which have a natural tendency to be effective in all types of cancer. Under the title—CIK cell therapy: past, present and future—he acknowledges the efforts made in 158 clinical trials involving patients treated with CIK cells (according to NLM), 17 phase III clinical trials (according to ClinicalTrials.gov), and certainly the satisfactory outcome of more than 5000 patients treated with CIK cells in clinical trials involving more than 30 tumor entities [4]. Since his lab and others have demonstrated that CIK cells are compatible with almost all kind of immune check point inhibitors, epigenetics drugs or commercial compounds, now with contemporary techniques like CAR-CIK therapy, it is reasonable to propose that cancer patients can express new hope for innovative treatment.

Next, *Prof. Xiubao Ren (China)* informed the audience about autologous CIK cell immunotherapy for lung cancer. In particular, he provided information about his multicenter, prospective, randomized, controlled phase II clinical trial of CIK cells in combination with chemotherapy for the treatment of advanced squamous cell carcinoma of the lung, in which eight major centers from China participated. He demonstrated that compared with chemotherapy alone (control group, *n* = 45), the median PFS and OS were prolonged by 4.7 months and 10.7 months, respectively, in the experimental group (autologous CIK cells in combination with chemotherapy, *n* = 45), which significantly reduced the incidence of disease progression and mortality in patients with advanced squamous cell carcinoma of the lung [5]. In addition, CIK cells in combination with chemotherapy significantly reduced the severe side effects caused by chemotherapy and improved patients' chemotherapy tolerance and quality of life. He then reported how the decision to include PD-1 inhibitors in the combination strategy of CIK cells helped to design a prospective single-arm phase IB trial for patients with stage IIIB-IV lung cancer. The team treated a total of 34 patients with lung cancer, with patients receiving first-line CIK cells in combination with PD-1 inhibitors and chemotherapy. Specifically, the results showed that ORR was 82.4%, DCR was 100%, and median PFS was 19.3 months. In patients with squamous cell carcinoma, the ORR was 73.7%, the DCR was 100%, and the median PFS was 17 months. In patients with non-squamous cell carcinoma, the ORR was 93.3%, the DCR was 100%, and the median PFS was not reached [6]. Compared with the results of the other phase III clinical trials, a better clinical benefit was obtained and there was no increased number of adverse events. On the basis of these findings, a multicenter, large-scale, randomized, controlled phase II clinical trial is currently being recruited, and further convincing results are expected. Besides, he shared some of the preclinical results, such as how CIK cell therapy partially reverses the DDP resistance of cisplatin-resistant A549/DDP lung cancer cells [7]. He also mentioned the observation that they focused on CD4+ T cells, an important subset of the CIK cell population, as they found that there was a significant difference in the proportion of these cells in CIKs from different patients by ex vivo expansion. Considering that this different proportion of CD4+ T cells might ultimately determine the ability of CIKs to kill tumor cells, they investigated how the CD4+ T subset induced phosphorylation of the AKT pathway by secretion of IL17A and upregulated the expression of T-bet/Eomes transcription factors, thereby restoring the function of CD8+/CD3+CD56+T cells and reversing the depletion of PD-1+Tim-3+T cells [8]. As for PD-1, his team experimentally confirmed that CIK in combination with PD1 inhibitors can improve efficacy in clinical trials [9]. Also, in the clinical setting, he highlighted a negative correlation between somatic copy number alterations (SCNA) and response to CIK+chemotherapy, suggesting SCNA as a predictive indicator in patients with lung adenocarcinoma treated with CIK+chemotherapy [10]. Similarly, he pointed that the coordinated suppression of HLA class II gene, checkpoint molecule expression and reduced immunosuppressive cell infiltration jointly contributed to the better efficacy of CIK treatment in lung SCC compared with lung adenocarcinoma [11].

The discussion continued with the presentation by *Prof. Antonio Rosato (Italy)*, who focused on the main results of ongoing projects aimed at exploring the therapeutic potential of retargeting CIK cells with clinical grade monoclonal antibodies (mAbs), Fc-engineered mAbs and bispecific antibodies (bsAb) to create a “universal” platform for adoptive cell therapy (ACT) without the need to genetically modify effector cells. He first recounted the results on retargeting against EGFR with cetuximab [12] and then discussed a modified expansion protocol for CIK cells [13]. He then pointed out that the unique effect of trastuzumab (TRS) and its optimized forms, as well as Her-2xCD3 bsAb, to be extremely powerful in enhancing the antigen-specific cytotoxicity of CIK cells against various Her-2+ cancer cell lines. Concerning the retargeting of CIK cells in hematologic malignancies, he shared initial thoughts on their hypothesis regarding the retargeting properties of clinical grade anti-CD20 mAbs [14] and how the optimized expansion protocol with blinatumomab (Blina, CD19xCD3) to generate CIK cells from patients was successfully transferred to the Laboratorio per le Terapie Cellulari Avanzate (LTCA) of the Hospital of Vicenza, adapted under GMP conditions and enabled the treatment of the first patient under the hospital exemption scheme in early 2023. Addressing cutting-edge technologies, he provided evidence from a comparative analysis of the therapeutic efficacy of retargeted CIK cells versus CAR-T, using the lentiviral vector for transduction and testing CAR-T cells in parallel with Tafa-targeted CIK cells. Conclusively, he demonstrated that retargeted CIK cells can lead to therapeutic efficacy comparable to that of CAR-T cells, thus providing an alternative approach for those patients who are ineligible for CAR-T therapy.

*Prof. Dario Sangiolo (Italy)* then delivered a presentation on CAR-CIK cellular immunotherapy against solid tumors and chemoresistant cancer stem cells. Besides discussing his earlier work on the successful use of CIK cells in a sarcoma tumor model, he also highlighted several lines of evidence for the potential utility of CAR-CIKs as a realistic cancer immunotherapy option. During his discussion of the major challenges in developing more effective immunotherapy strategies (especially involving cancer stem cells, which are a potential cause of chemoresistance and disease relapse), he emphasized that promising results can be expected from CIK cell-based approaches.

*Prof. Dr. Zibing Wang (China)* also presented the results of the clinical trials conducted by his team on CIK cells for the treatment of renal, pancreatic, and lung cancers. Referring to their experience in initially evaluating the efficacy of CIK cells as a stand-alone treatment for metastatic renal cell carcinoma (RCC), [15]. While describing that in the controlled clinical trial of 29 patients who received approximately 5 billion CIK cells and 2 million IU IL-2 per day for five consecutive days in each cycle, four patients achieved partial response (PR), 18 achieved stable disease (SD), and seven achieved disease progression (PD), with no complete response (CR) observed. In addition, the 1-year survival rate was 83%. Interestingly, they found that RCC patients had higher levels of myeloid-derived suppressor cells (MDSC) in their peripheral blood compared to healthy volunteers and that after infusion of CIK cells, MDSC levels decreased. Thus, suggesting that MDSC could potentially be used as a marker for predicting the prognosis of RCC patients receiving treatment with CIK cells. Consequently, they hypothesized that inhibition of MDSCs might increase the efficacy of CIK cells, and they conducted a series of studies to investigate whether MDSCs could be effectively suppressed by gemcitabine and fluorouracil [16]. They enrolled 53 patients (metastatic renal cancer: *n* = 17, advanced pancreatic cancer: *n* = 10, metastatic melanoma: n = 26) into the study and divided them into two groups: one group treated with CIK cells alone and the other group receiving CIK cells in combination with chemotherapy. The results showed that the combination of CIK cells and chemotherapy increased 1-year survival and median survival in patients with metastatic kidney and pancreatic cancer. Similarly, they confirmed that the combination of CIK cells with chemotherapy can also increase the anti-tumor efficacy in pancreatic cancer [17]. He concluded that the addition of MDSC-reducing chemotherapy to CIK cell therapy improved survival in patients with metastatic renal and pancreatic cancer. In the special focus on PD-1, his team also studied two cases in which CIK cells combined with PD-1 resulted in a partial response [18]. The effectiveness of PD-1 is known to depend solely on the level of PD-L1 expression, which serves as a biomarker. However, in two cases his team investigated, the tumor had low or undetectable PD-L1 expression, as well as low tumor mutation burden and MSS status. Thus, suggesting that combining CIK cells with PD-1 could significantly increase the number of individuals who may benefit from immunotherapy. His team observed similarly encouraging results in a recent clinical trial of 29 patients combining CIK cells with PD-1 in the treatment of renal cancer [19], 125 patients with non-small cell lung cancer with EGFR wild-type lung adenocarcinoma who were tested for chemotherapy alone and CIK cells in combination with chemotherapy [20], and patients with small cell lung cancer received first-line chemotherapy (etoposide plus platinum) in combination with CIK cells [21]. In conclusion, he mentioned that his team also used Retronectin-activated CIK cells [22] because they have a higher percentage of CD3+CD4+cells and CD3+CD28+cells compared with other types of CIK cells.

Continuing the next session, *Dr. Sarah Tettamanti (Italy)* presented the culmination of a decade-long research effort conducted by the Tettamanti Center in Monza in collaboration with researchers in Bergamo (Papa Giovanni XXIII Hospital). Their work focused on improving the efficacy of CIK cells against hematologic malignancies by engineering them to express Chimeric Antigen Receptors (CARs) by using the non-viral Sleeping Beauty transposon system. She emphasized that this particular approach has several advantages over viral systems, including cost efficiency due to the use of non-viral plasmids, a nonimmunogenic profile, and a near-random integration pattern that reduces the risk of genotoxicity. In addition, she reported encouraging results from a successful clinical trial, a Phase I-IIa dose-escalation study evaluating the use of CARCIK-CD19 produced in donor cells using the SB Transposon system (NCT03389035). The objective of the study was to evaluate the safety and efficacy of this approach in patients with B-cell acute lymphoblastic leukemia (B-ALL) who had relapsed after hematopoietic stem cell transplantation (HSCT). Intriguingly, the study showed promising results for CARCIK-CD19 therapy, with encouraging complete response rates and acceptable safety profiles. Notably, 73% of patients receiving the highest dose achieved a complete response at day 28. The 6-month overall survival (OS) rate was 73%, and the OS rate at 1 year was 48% in patients receiving the highest CAR-CIK cell dose. Minimal residual disease (MRD) was negative in 8 of 11 patients with complete response. The safety profile was satisfactory, with cytokine release syndrome (CRS) observed in some patients and grade 3 immune effector cell-associated neurotoxicity syndrome (ICANS) occurring in two patients. In addition, no secondary graft-vs-host disease (GVHD) was observed. In the second part of her presentation, she reviewed recent improvements to the non-viral CAR-CIK platform, including the use of next-generation SB plasmids (hyperactive SB100X transposase and pT4 transposon), shortening of cell culture time from 21–28 to 14–16 days, eliminating the need for irradiated feeder cells after nucleofection, and the introduction of the G-REX bioreactor instead of standard T-flasks. She pointed out that this refined production process will be tested in an upcoming Phase I-II trial to evaluate the safety of allogeneic CARCIK-CD19 in adult and pediatric patients with relapsed/refractory B-cell non-Hodgkin’s lymphoma (R/R B-NHL). In addition, she explained the application of this non-viral Sleeping Beauty platform to produce an “off-the-shelf” CAR-CIK cell therapy from cord blood (CB). Here, the use of IL7/IL15 cytokines as opposed to conventional IL-2 resulted in a higher central/naïve memory phenotype and more fold expansion when starting from thawed CB bags. In closing, she addressed the application of CAR-CIK cells to combat acute myeloid leukemia (AML) and explained how her team is currently working to characterize a trans-signaling dual CAR designed specifically for CD123 and CD33 target antigens. Dual CAR-CIK cells demonstrated potent antileukemic activity while mitigating potential on-target off-tumor toxicities. In addition, they developed armored CAR-CIK cells carrying CD33CAR and CXCR4, which exhibited better homing abilities in the bone marrow niche compared with single CD33CAR-engineered CIK cells.

*Prof. Yi Zhang (China)* then spoke about CIK-based cell therapy for solid tumors. He talked about his team's experience and efforts in conducting clinical trials to treat patients with melanoma, renal cell carcinoma, squamous cell carcinoma of the esophagus, non-small cell lung cancer and colorectal carcinoma with CIK cells. He indicated that melanoma patients in early stages, i.e., stage I or II, but not stage II or IV, can benefit from adjuvant CIK cell therapy. He also mentioned that in clinical trials, CIK cells were able to prolong OS and PFS in the above-mentioned patients enrolled in his center, without any severe side effects. He also informed that cord blood (CB) derived mononuclear cells could be another resource for producing better CIK cells in terms of proliferation ability, chemotherapy resistance, higher production of pro-inflammatory cytokines and the ability to trafficking to the tumor by expressing higher levels of CXCR3, CCR6 and CCR7 on the surface of CB-CIK cells.

On an interesting note, next *Prof. Eva Rettinger (Germany)* addressed how development and clinical translation can be facilitated by in-vitro expansion in the presence of interleukin (IL)-15, which can significantly increase the antileukemic cytotoxicity and thus the *in-vitro* manufacturing time of CIK cells. She presented information on CIK-FFM products used between 2016 and 2021 in a multicenter, prospective, phase 1/2 clinical trial (FFM—CIK-Cell Study 01 study, Eudra-CT: 2013-005446-11) as advanced therapy medicinal products (ATMPs) in children and adults with impending relapse of hematologic malignancies after allogeneic hematopoietic stem cell transplantation (HSCT). Interestingly, the preclinical and clinical data confirmed that CIK-FFM products can be manufactured for clinical use and are well tolerated without significantly affecting the rate of graft-versus-host disease (GVHD). Thus, suggesting that CIK-FFM products can be used to eradicate molecular disease and consolidate after transplantation, and provide palliative care for recurrent hematologic malignancies. Overall, she envisions that this will support the initiation of a multicenter, prospective, phase 3 clinical trial to treat pediatric AML patients at risk of relapse after allogeneic HSCT with a preventive or prophylactic CIK-FFM approach. Intriguingly, she emphasized that CIK-FFM cells can be engineered in-vitro with a second-generation CAR targeting, for example, the tumor-associated surface antigen ErbB2 (HER2/neu), a therapeutically useful target found in rhabdomyosarcoma (RMS) and other solid tumor entities. Her team used lentiviral vectors (LV) for CAR gene transfer into CIK cells, which resulted in robust expression of the respective CAR. ErbB2-CAR-modified CIK cells exhibited sufficient in-vitro expansion and retained NKG2D-mediated cytotoxicity against ErbB2-negative tumors, whereas ErbB2-CAR CIK cells, but not parental CIK cells, proliferated, infiltrated, and efficiently lysed ErbB2-positive tumor cell monolayers and 3D tumor spheroids in-vitro. Moreover, in an experimental metastasis model in NSG mice, ErbB2-CAR-CIK cell therapy inhibited the engraftment and proliferation of RH30^GFP/Luc^ cells derived from the bone marrow metastasis of a 17-year-old male with alveolar RMS, carrying a p53 mutation and expressing the Pax3/FKHR fusion protein. A single administration of ErbB2-CAR-CIK cells allowed settlement, infiltration, persistence, and spread at tumor sites, suggesting that ErbB2-CAR-CIK therapy was able to eliminate tumor cells by eliciting long-lasting responses from the innate and adoptive immune systems in addition to the CAR molecule. She therefore concluded that CAR-CIK cell therapy may be beneficial in resistant tumors such as RMS with heterogeneous, low CAR target expression, warranting a more detailed evaluation of this approach in the future.

In the last session, young investigator *Dr. Kevin Aaron Fenix (Australia)* spoke about his interest in developing novel prognoses and treatments for colorectal cancer (CRC). In particular, he shared how his team recently came across the utility of CIK cell therapy for the clinical treatment of metastatic colorectal cancer. He explained how, while working on their recent systematic review of the clinical utility of CIK cell therapy in the clinical treatment of colorectal cancer, they became aware of potential appropriate strategies [23]. In fact, his team was able to successfully translate those to CRC patient populations, which he shared with the audience by showing some unpublished results. Of interest were the data on the development of a GMP-compatible cell culture protocol for potential CIK cell therapy in metastatic colorectal cancer patients in South Australia. In conclusion, he emphasized the urgent need to standardize protocols for CIK cell generation (culture conditions, quality assurance practices, blood collection, infusion protocols, and combination with other therapies) to achieve greater acceptance of this unique therapy.

Closing the meeting, Prof. Schmidt-Wolf wrapped up his speech by stating that while CIK cell therapy has gone through many trials and tribulations over the past 30 years, by now there is a plethora of knowledge about its clinical performance. Undeniably, the results of patients treated with CIK cell therapy have been very beneficial, not just in one type of cancer, but in multiple types. In light of what we have learned, it is certain that the combination of adoptive immune cells, inhibitors (/immune checkpoint/epigenetic/targeted inhibitors), and traditional therapy can help to defeat cancer. Lastly, some important announcements were made: (1) a standardized protocol for CIK cell generation, (2) the establishment of the ISCC (International Society of CIK Cells), (3) a stronger CIK cell network integrating preclinical research and clinical trials, (4) future annual meetings, and (5) a common repository under the umbrella of a common homepage/website. Please contact the corresponding author if you are interested in becoming a member of the ISCC.

## Data Availability

Not applicable.

## Abbreviations

CIK cells: Cytokine-induced killer cells
CRC: Colorectal cancer
ISCC: International society of CIK cells
NSCLC: Non-small-cell lung cancer

## References

[1] Sharma A, Schmidt-Wolf IGH (2021). 30 years of CIK cell therapy: recapitulating the key breakthroughs and future perspective. J Exp Clin Cancer Res.

[2] Schmidt-Wolf IG, Negrin RS, Kiem HP, Blume KG, Weissman IL (1991). Use of a SCID mouse/human lymphoma model to evaluate cytokine-induced killer cells with potent antitumor cell activity. J Exp Med.

[3] Schmidt-wolf IGH, Finke S, Trojaneck B, Denkena A, Lefterova P, Schwella N (1999). Phase I clinical study applying autologous immunological effector cells transfected with the interleukin-2 gene in patients with metastatic renal cancer, colorectal cancer lymphoma. Br J Cancer.

[4] Zhang Y, Schmidt-Wolf IGH (2020). Ten-year update of the international registry on cytokine-induced killer cells in cancer immunotherapy. J Cell Physiol.

[5] Liu L, Gao Q, Jiang J (2020). Randomized, multicenter, open-label trial of autologous cytokine-induced killer cell immunotherapy plus chemotherapy for squamous non-small-cell lung cancer: NCT01631357. Signal Transduct Target Ther.

[6] Zhou Li, Xiong Y, Wang Y (2022). A phase IB trial of autologous cytokine-induced killer cells in combination with sintilimab, monoclonal antibody against programmed cell death-1, plus chemotherapy in patients with advanced non-small-cell lung cancer. Clin Lung Cancer.

[7] Yang L, Chunjuan Du, Lei Wu (2017). Cytokine-induced killer cells modulates resistance to cisplatin in the A549/DDP cell line. J Cancer.

[8] Liu S, Meng Y, Liu L (2022). CD4+ T cells are required to improve the efficacy of CIK therapy in non-small cell lung cancer. Cell Death Dis.

[9] Liu S, Meng Y, Liu L (2023). Rational pemetrexed combined with CIK therapy plus anti-PD-1 mAbs administration sequence will effectively promote the efficacy of CIK therapy in non-small cell lung cancer. Cancer Gene Ther.

[10] Kou F, Lei Wu, Zhu Ye (2022). Somatic copy number alteration predicts clinical benefit of lung adenocarcinoma patients treated with cytokine-induced killer plus chemotherapy. Cancer Gene Ther.

[11] Wang J, Yang F, Sun Q (2021). The prognostic landscape of genes and infiltrating immune cells in cytokine induced killer cell treated-lung squamous cell carcinoma and adenocarcinoma. Cancer Biol Med.

[12] Sommaggio R, Cappuzzello E, Dalla Pietà A, Tosi A, Palmerini P, Carpanese D, Nicolè L, Rosato A (2020). Adoptive cell therapy of triple negative breast cancer with redirected cytokine-induced killer cells. Oncoimmunology.

[13] Palmerini P, Dalla Pietà A, Sommaggio R, Ventura A, Astori G, Chieregato K, Tisi MC, Visco C, Perbellini O, Ruggeri M, Cappuzzello E, Rosato A (2020). A serum-free protocol for the ex vivo expansion of cytokine-induced killer cells using gas-permeable static culture flasks. Cytotherapy.

[14] Dalla Pietà A, Cappuzzello E, Palmerini P, Ventura A, Visentin A, Astori G, Chieregato K, Mozzo V, Perbellini O, Tisi MC, Trentin L, Visco C, Ruggeri M, Sommaggio R, Rosato A (2021). Innovative therapeutic strategy for B-cell malignancies that combines obinutuzumab and cytokine-induced killer cells. J Immunother Cancer.

[15] Wang Z, Zhang Y, Liu Y, Wang L, Zhao L, Yang T, He C, Song Y, Gao Q (2014). Association of myeloid-derived suppressor cells and efficacy of cytokine-induced killer cell immunotherapy in metastatic renal cell carcinoma patients. J Immunother.

[16] Wang Z, Liu Y, Zhang Y, Shang Y, Gao Q (2016). MDSC-decreasing chemotherapy increases the efficacy of cytokine-induced killer cell immunotherapy in metastatic renal cell carcinoma and pancreatic cancer. Oncotarget.

[17] Wang Z, Liu Y, Li R, Shang Y, Zhang Y, Zhao L, Li W, Yang Y, Zhang X, Yang T, Nie C, Han F, Liu Y, Luo S, Gao Q, Song Y (2016). Autologous cytokine-induced killer cell transfusion increases overall survival in advanced pancreatic cancer. J Hematol Oncol.

[18] Wang Z, Liu X, Till B, Sun M, Li X, Gao Q (2018). Combination of cytokine-induced killer cells and programmed cell death-1 blockade works synergistically to enhance therapeutic efficacy in metastatic renal cell carcinoma and non-small cell lung cancer. Front Immunol.

[19] Zhao L, Li T, Song Y, Yang Y, Ma B, Zhang Y, Shang Y, Xu B, Guo J, Qin P, Han L, Fu X, Lin H, Liu L, Ren X, Wang Z, Gao Q (2022). High complete response rate in patients with metastatic renal cell carcinoma receiving autologous cytokine-induced killer cell therapy plus anti-programmed death-1 agent: a single-center study. Front Immunol.

[20] Zhao L, Li W, Wang Z, Yang Y, Zhang Y, Shang Y, Ren X, Gao Q (2018). Survival benefit from RectroNectin-activated cytokine-induced killer cells combined with chemotherapy in advanced EGFR wild-type lung adenocarcinoma. Immunotherapy.

[21] Ma B, Zhou Y, Shang Y, Zhang Y, Xu B, Fu X, Guo J, Yang Y, Zhang F, Zhou M, Huang H, Li F, Lin H, Zhao L, Wang Z, Gao Q (2022). Sintilimab maintenance therapy post first-line cytokine-induced killer cells plus chemotherapy for extensive-stage small cell lung cancer. Front Oncol.

[22] Han L, Shang YM, Song YP, Gao QL (2016). Biological character of retronectin activated cytokine-induced killer cells. J Immunol Res.

[23] Li CMY, Tomita Y, Dhakal B, Li R, Li J, Drew P, Price T, Smith E, Maddern GJ, Fenix KA (2023). Use of cytokine-induced killer cell therapy in patients with colorectal cancer: a systematic review and meta-analysis. J Immunother Cancer.

